# Disorder Type and Severity as Predictors of Mental Health in Siblings of Children with Chronic Disorders

**DOI:** 10.1007/s10803-025-06771-6

**Published:** 2025-02-25

**Authors:** Trude Fredriksen, Stian Orm, Caitlin M. Prentice, Solveig Kirchhofer, Erica Zahl, Matteo Botta, Torun M. Vatne, Krister W. Fjermestad

**Affiliations:** 1 Innlandet Hospital Trust, Po Box 104, 2381 Brumunddal, Norway; 2 Department of Psychology, University of Oslo, Forskningsveien 3a, 0317 Oslo, Norway; 3 Frambu Resource Centre for Rare Disorders, Sandbakkvn 18, 1404 Siggerud, Norway; 4 Inland Norway University of Applied Sciences, Vormstuguvegen 2, 2624 Lillehammer, Norway; 5 Nic Waal Institute, Child and Adolescent Mental Health Services, Lovisenberg Hospital Trust, Nydalen, P Box N-49-70, Oslo, Norway

**Keywords:** Children with chronic disorders, Diagnoses, Disorder severity, Sibling internalizing and externalizing problems

## Abstract

Siblings of children with chronic disorders are at risk of developing mental health problems. Studies are inconclusive about whether sibling mental health is best predicted by the specific diagnoses of the child with disorder or by transdiagnostic factors. The aims of the present study were (1) to examine if specific diagnoses predicted sibling mental health, and (2) to examine if disorder severity in the child with the chronic disorder predicted sibling mental health. Baseline data from a randomized controlled trial were used. Siblings (aged 8 – 16 years) of children with chronic disorders and their parents were recruited from eight municipality and hospital clinics (N = 288). The children with chronic disorders were placed in ICD-10 diagnostic categories based on combined parent report and clinical records. Regression analyses with the most frequent primary diagnoses (ADHD, Asperger syndrome, autism, down syndrome, rare disorders) and a measure of disorder severity as predictors of sibling mental health were run. Father-reported disorder severity predicted sibling-reported internalizing problems and father-reported internalizing and externalizing problems in siblings. Mother-reported disorder severity predicted mother-reported sibling internalizing problems. No single primary diagnosis predicted sibling internalizing or externalizing problems. Disorder severity does to an extent predict sibling mental health, whereas single diagnostic categories do not. Disorder severity may be used to identify siblings at risk and/or in need of interventions. Fathers should be included in assessment and health care for siblings as their reports seem to predict sibling mental health better than the mothers.

## Disorder Type and Severity as Predictors of Mental Health in Siblings of Children with Chronic Disorders

Siblings of children with a chronic disorder (herein; “siblings”) are affected by growing up in a family with a child with a chronic disorder (Blamires et al., 2024; Pinquart, 2023; Wolff et al., 2022). Chronic disorders are intellectual and/or physical conditions that persist over time and require ongoing monitoring and management, often across the lifespan. The etiology of chronic disorders is understood as a combination of genetic-biological factors, psychosocial-behavioral factors, and social-environmental factors (Perrin et al., 1993). Reviews and meta-analyses have shown disadvantageous consequences for siblings of children with a chronic disorder, including poorer wellbeing (Mariñez et al., 2022; Martinez et al., 2022). Siblings tend to undertake a caregiver role and take on more responsibilities than siblings of children without a chronic disorder (Baker & Claridge, 2023; Hilario, 2022). Furthermore, siblings may experience differential parental treatment and may worry about the child with chronic disorder and/or their parents (Kelada et al., 2022). Unpredictable and frightening incidents related to the chronic disorder, such as epileptic seizures, sudden emotional outbursts, or violent behavior, may lead to fear and elevated stress (Lummer-Aikey & Goldstein, 2021). Hence, siblings are at increased risk of both internalizing problems, such as depression and anxiety, and externalizing problems, such as oppositional and aggressive behavior (Mariñez et al., 2022; Martinez et al., 2022).

Although siblings are at increased risk of poorer psychological functioning than their peers, studies also show that being the sibling of a child with a chronic disorder may be associated with positive outcomes like prosocial skills, greater acceptance, and maturity (Blamires et al., 2024; Brolin et al., 2024; Wolff et al., 2022). A recent meta-analysis of studies on siblings of children with a chronic physical chronic disorder found that although siblings had elevated levels of internalizing and externalizing problems, they also displayed higher levels of prosocial behavior than siblings of children without physical health conditions (Pinquart, 2023). Another review comprising studies on siblings of children physical and mental health diagnoses reported inconsistent findings, with either an increased risk of mental health problems or no difference in risk for siblings (Martinez et al., 2022). Hence, the findings regarding mental health outcomes in siblings are conflicted, and it is unclear which siblings are at a greater risk and may need intervention. The current study aims to contribute to our knowledge regarding which siblings should be in focus for health professionals and be prioritized for interventions.

The evidence for effective sibling interventions remains limited (Mitchell et al., 2021; Wolff et al., 2023). Current interventions primarily focus on psychoeducation and support groups, with variable outcomes (Hartling et al., 2014; Marquis et al., 2023). Recent efforts have shifted towards a systemic approach targeting parents as well as the siblings themselves, yet challenges persist in identifying the most effective interventions; if interventions should be tailored to specific diagnoses or directed towards shared challenges of having a child with a chronic disorder in the family (Mitchell et al., 2021; Wolff et al., 2023). An essential step towards establishing effective interventions is to identify factors that predict siblings’ mental health. Chronic disorders encompass multiple diagnoses, and many sibling studies comprise children with a range of chronic disorders (Pinquart et al., 2023; Wolff et al., 2022). Differentiating between diagnostic categories may still be important in designing effective interventions or services for siblings. For example, a sibling of a child with cerebral palsy may need different types of interventions than siblings of children with ADHD. The everyday life of a sibling of a child cerebral palsy may be characterized by challenges including an extensive need for physical assistance, and cerebral palsy is also a clearly visible functional impairment, while in the daily life of the sibling of a child with ADHD the challenges may not be as visible for those outside the family but be characterized by frequent emotional and behavioral outbursts. Hence, studies with samples representing different diagnostic categories are advantageous if they allow for comparisons between chronic disorders. However, most studies on siblings do not have a large enough sample size to allow such analyses (Tudor et al., 2015). Thus, there is a lack of well-powered studies comparing the impact of different chronic disorders on sibling outcomes, and such studies are needed to help solve the current ambiguities between previous studies.

Much research has a transdiagnostic approach, as sibling studies show that there is more variability of mental health problems within than between diagnostic categories (of the child with a chronic disorder) (Stein & Jessop, 1989). Transdiagnostic factors like behavioral and emotional problems related to the chronic disoder and invasiveness of treatment regimens seems to be more influential than the diagnosis itself (Sharp & Rossiter, 2002; Vermaes et al., 2012; Wolff et al., 2023). However, some studies have found that siblings of children with autism spectrum disorders have more adjustment problems than those of other diagnostic groups, such as siblings of children with Down syndrome (Hodapp & Urbano, 2007) or compared to all other siblings in a population-based cohort study (Jokiranta-Olkoniemi et al., 2016). One study found that siblings of children with cerebral palsy, hematologic/oncologic disease, or asthma had poorer psychosocial health scores compared to a control group of siblings of children with no chronic disorder, but that siblings of children with type 1 diabetes, epilepsy and celiac disease did not report worse psychosocial health than controls (Dinleyici et al., 2020). A study that compared siblings of children with physical illness with siblings of children with physical and mental comorbidity, found no significant differences regarding sibling mental health comparing these two groups when controlling for parental and family factors (e.g., marital status, education, and income) (Qureshi et al., 2023). Due to the limited number of studies in the field, there is not yet sufficient evidence to conclude regarding the role of different chronic disorder categories for siblings’ mental health. The current study aims to help build this evidence-base.

In the current study we examine whether specific diagnostic categories predict mental health better than other diagnostic categories, and whether a transdiagnostic measure of chronic disorder severity is a better predictor than single diagnostic categories. Disorder severity is here used as a term for a general functional impairment in the daily lives of the child with the chronic disorder. This implies lack of age appropriate self-help skills (e.g., bites and chews on things, urinates outside toilet, runs away, takes off clothes and needs help to get dressed), emotional problems (e.g., shy, anxious, afraid of specific things and situations, getting overexcited), behavior problems (e.g., swears, hits or kicks others, steals food), and problems with communication and social relating (e.g., does not respond to other people’s feelings, avoids eye contact, uncritical in contact with strangers or other people in general, talking to oneself).

We have two primary research questions: (1) Are different diagnostic categories associated with varying levels of sibling mental health? (2) Does parent-reported chronic disorder severity predict sibling mental health? The first question was exploratory as existing studies have shown mixed results and few studies have been sufficiently powered to compare sibling mental health with the different chronic disorders. For the second question, we expected that parents’ perceived symptom severity in the child with a chronic disorder would be a significant predictor of sibling mental health (Schumann et al., 2024; Vermaes et al., 2012; Wolff et al., 2023). Studies show findings in different directions regarding age and gender as predictors of mental health in siblings. Some studies suggest that it is more challenging to be an adolescent or older than the child with the diagnosis, while others show the opposite (Deavin et al., 2018). Findings in some studies indicate that sisters are more at risk than brothers for experiencing mental health issues, while others suggest that brothers face more challenges (Boyle et al., 2023; Hilario, 2022; Lavoie et al., 2019). Therefore, this study controls for gender and age. This study focus on characteristics of the child with a chronic disorder and impact on sibling mental health. However, as family economy and mothers´ and fathers´ education level are known impact factors on children´s mental health, these variables were also examined in this study.

## Methods

### Participants, Setting and Procedures

In the current study we utilized baseline data from an ongoing randomized controlled trial (SIBS-RCT) evaluating the effects of an intervention on siblings (*N* = 288) and their parents where the primary outcome was sibling mental health (Fjermestad et al., 2020). Families were recruited through municipal and specialist health service centers across Norway. The eligibility criteria included being the sibling (8 – 16 years) of a child diagnosed with a chronic disorder (0—18 years) who received specialist and/or municipal health services. See Table 1 for demographic information on the participants.

**Table 1:** Participant Demographic Information (N = 288)

		Mean	SD	Range	Percent
Siblings’ age (years)		10.5	2.1	8–16	
Child with diagnosis’ age		10.5	3.4	3–18	
Mothers’age		42.3	5.4	28–54	
Fathers’age		44.9	5.8	31–66	
Siblings’ gender					
Girls					55.2
Boys					44.8
Child with diagnosis’ gender					
Girls					36.6
Boys					63.4
Family economic situation					
Very poor					0.4
Poor					6.4
Neutral					28.9
Good					51.1
Very good					13.2
Family constellation					
Living with both parents					80.4
Mostly with mother					17.9
Mostly with father					1.3
With other caretakers					0.4
Ethnicity					
European					91.9
Asian					2.4
African					2.4
Other					3.3

Parents gave written informed consent to participation and retrieval of diagnostic information from the health care services. Children (with a chronic disorder) over the age of 16 years who the parents considered to be competent to provide informed consent, gave consent on their own behalf. They were all informed that participation was voluntary. The study was approved by the Regional Committees for Medical and Health Research Ethics.

## Measures

### The Strengths and Difficulties Questionnaire (SDQ)

The 25-item the Strengths and Difficulties Questionnaire (SDQ) (Goodman et al., 1997) was used to measure sibling mental health. Siblings and parents rated statements related to emotional, conduct, hyperactivity-attention, and peer problems on a 3-point Likert scale from 0 (*not true*) to 2 (*certainly true*) (e.g., *“I am restless, I cannot stay still for long*”). Originally the SDQ comprises five subscales. In this study, we looked at the four difficulties subscales that were summed into internalizing and externalizing composite scores. The internalizing scale comprises the Emotional Problem Scale and the Peer Problem Scale. The externalizing scale comprises the Conduct Problem Scale and the Hyperactivity-inattention Scale. The internalizing and externalizing composites were used in place of the five subscales because siblings represent a non-clinical sample (Goodman et al., 2010). Adequate test–retest reliability, concurrent validity, and the ability to distinguish between community and clinical samples has been reported for the SDQ (Goodman, 2001; Goodman & Scott, 1999). In the current study, internal consistency for the SDQ was satisfactory (sibling α = 0.72; mother α = 0.72; father; α = 0.71). British norms for the SDQ (2013, March 22) available at sdq.info.org (https://www.sdqinfo.org/g0.html) were used to determine how the siblings’ scores in this population were compared to clinical cut off.

### The Developmental Behavior Checklist the Parent/Carer Version (DBC-P)

The 96-item Developmental Behavior Checklist Primary Carer Version (DBC-P) (Einfeld & Tonge, 1995) was used to measure parent perceived symptom severity in the child with chronic disorders. Parents rated symptoms in children on a 3-point Likert scale 0 (*not true as far as you know)* to 2 (*very true or often true*) (e.g., “*Refuses to go to school, activity center, or workplace, Repeats the same word or phrase over and over*”). The DBC-P comprises five subscales: disruptive/antisocial, self-absorbed, communication disturbance, anxiety, and social-relating. The scores on each subscale can be added into a total score indicating a global expression for the symptom severity of the child with a chronic disorder. The DBC-P was developed to uncover difficulties in children with intellectual and communicational deficiency. It assesses difficulties across a broad range in children with intellectual and communicative challenges, e.g., the extent of deviant and disruptive behavior, how they relate socially to others, their level of interest in others, how they express themselves and their desire for communication with others, and the child's independence—both emotionally and practically. The DBC-P has shown satisfactory reliability and validity. Hence, as the sample in the current study comprised children with diagnoses which both do and do not involve intellectual deficiency, the internal validity was checked for all diagnostic categories separately. Adequate test–retest reliability for both total scale score and for scores on each of the subscales, concurrent and discriminative validity have been reported for the DBC-P (Einfeld & Tonge, 1995). Acceptable Cronbach’s alphas (> 0.70) were found for all the diagnostic categories. In the current study, internal consistency for the total DBC-P score was satisfactory (mothers α = 0.94; fathers α = 0.94).

### Diagnostic Information

Diagnoses of the children with chronic disorders were reported by parents (N = 288). About half of the diagnoses (N = 155) were confirmed with information from the clinic where the child was examined and diagnosed. Where the diagnostic information given by the parents differed from the clinic information, the clinic information was given priority. The few cases where there was an incongruence (2.8%) were discussed and decided upon by two child- and adolescent psychologist based on all available information about the family. The remaining 133 children were not possible to trace in the health care system because they had not provided information about where the child was diagnosed, or the diagnostic information was not available in the child’s records. These were mainly participants recruited through municipality health care centers, which provide health care services but do not diagnose the conditions included in this study. The diagnostic categories (Table 2) were established based on ICD-10 (International Statistical Classification of Diseases and Related Health Problems 10th Revision; World Health Organization, 2004). The division between autism and Asperger syndrome was done because the diagnostics of the children with a chronic disorder were done according to the ICD-10 manual which separate the autism spectrum disorders into categories. The separation of the two labels autism and Asperger syndrome was reflected in the information given by both parents and the clinics.

**Table 2:** Distribution of diagnosis in the sample and primary diagnoses (N = 288)

Total distribution of diagnoses			Total distribution of primary diagnoses	
	N	Percent	N	Percent
Attention deficit hyperactivity disorder	97	33.6	**75**	**26.0**
Rare disorder	52	18.0	**52**	**18.0**
Emotional and conduct disorder	47	16.3	10	3.5
Autism	46	15.9	**43**	**14.9**
Asperger syndrome	35	12.1	**34**	**11.8**
Intellectual disability disorder	32	11.1	7	2.4
Somatic disorder	30	10.4	3	1.0
Down syndrome	29	10.0	**29**	**10.0**
Tourette’s syndrome	28	9.7	14	4.8
Eating disorders	15	5.2	13	4.5
Cerebral palsy	11	3.8	7	2.4
Specific developmental disorders	11	3.8	2	0.7
One diagnosis	167	57.8		
Two diagnoses	89	30.8		
Three diagnoses	32	11.4		

All diagnostic categories presented according to frequency and primary diagnoses presented in the third and fourth column with the most frequent diagnostic categories ($\ge$ 10%) in bold

### Data Analysis

The frequencies of the various diagnoses were calculated according to categorization in ICD-10. Mean scores and standard deviation for every diagnostic category, independent of whether the diagnoses were primary, secondary, or tertiary, were computed for all SDQ scores (siblings’ internalizing and externalizing problem scores and mothers’ and fathers’ reports about siblings` internalizing and externalizing scores). We examined descriptive statistics and distribution of diagnoses in the sample based on combined information from parents and the clinics. Linear regression analysis with family economy and mothers´ and fathers´ education level as predictors for all the outcome variables were ran. Linear regression analyses were run with all diagnostic categories as predictors for the SDQ scores (sibling, mother, and father report about internalizing and internalizing problems). All the diagnostic categories were coded with dummy variables (0,1). Deviation coding were used for all diagnostic categories to compare each diagnostic category with the samples grand mean (across diagnostic categories). A sensitivity power analysis showed that our sample achieved a power of 0.80 to detect a small effect size of *R*^2^ = 0.08 in the most complex model with nine predictors and the smallest sample size of *n* = 200 (Faul et al., 2007, 2009). However, due to some very small diagnostic categories and skewed distribution, linear regressions were then run with the most frequent *primary* diagnostic categories (ADHD, Asperger Syndrome, autism, Rare Disorders) as predictors for SDQ scores (siblings, mothers, and fathers) to check whether having a diagnosis as primary would be a significant predictor. We controlled for disorder severity (DBC-P), sibling gender and age in all regression models. IBM SPSS 27 was used for all analyses.

## Results

The distribution of diagnoses is shown in Table 2 listed from the most frequent to the last frequent diagnostic category, together with the percentage of primary diagnoses in every diagnostic category. Information about the proportion of children with one, two and three diagnoses, respectively, is also shown. Twelve diagnostic categories were identified. Rare disorders (e.g., Cornelia de Lange Syndrome, Fragile X Syndrome, Kearn Sayres syndrome, Kleeftras Syndrome) are grouped together as they are all diagnosed under the same chapter (XVII Congenital malformations, deformations, and chromosomal abnormalities) in ICD-10 and in this sample each of the rare disorders was mostly represented by one person except from three of the conditions (Cardiofaciocutaneous syndrome (2), DiGeorge syndrome (6) and Prader-Willi syndrome (2)). Hence, it was not appropriate to separate these disorders into several categories. Emotional and conduct disorders were also grouped together as they fall under the category of two etiologically closely related conditions (anxiety, obsessive–compulsive disorder, post-traumatic stress disorder, bipolar affective disorder, depression). All additional diagnoses were coded as secondary or third together with the primary diagnoses.

We examined the average scores on the SDQ internalizing and externalizing composite scores for siblings, mothers, and fathers according to all diagnostic categories independent of whether the diagnoses were given as primary, secondary, or tertiary. Means and SDs are shown in Table 3. When compared to British norms, a parent score between 4 and 7 and self-score between 5 and 8 on internalizing problems scales are classified as “slightly raised”. For externalizing problems, a parent score between 8 and 10 and self-reported score between 6 and 10 are classified as “slightly raised” (Goodman & Goodman, 2009). Siblings reported internalizing problems within this range when the child with chronic disorder had autism, emotional and/or conduct disorders and cerebral palsy. Mothers’ report of sibling internalizing problems was within this range for all diagnostic categories. Fathers’ report of sibling internalizing problems was within this range for all diagnostic categories except somatic disorders and eating disorders. Siblings’ report about externalizing problems was within the “slightly raised” range when the child with chronic disorders had ADHD, Tourette’s syndrome, or cerebral palsy. None of the parents’ reports on sibling externalizing problems were within the “slightly raised” range.

**Table 3:** Mean scores on internalizing and externalizing Strengths and Difficulties Questionnaire for siblings reported by both siblings and parents distributed on all diagnostic categories

	Siblings				Mothers				Fathers			
	Internalizing		Externalizing		Internalizing		Externalizing		Internalizing		Externalizing	
	Mean	SD	Mean	SD	Mean	SD	Mean	SD	Mean	SD	Mean	SD
Diagnoses												
ADHD	**4.93**	3.55	6.12*	3.84	5.04*	4.11	**5.60**	4.90	4.78*	3.67	4.57	4.53
RD	4.61	3.22	4.84	2.97	4.67*	4.21	4.30	3.05	4.88*	3.14	3.84	2.64
AUT	5.38*	2.72	5.69	3.61	5.53*	3.25	**5.53**	3.68	5.55*	2.93	4.25	2.67
EMD	5.44*	3.46	5.42	2.72	5.20*	3.68	4.10	3.19	5.25*	3.19	3.83	3.55
SD	4.75	2.92	5.80	3.37	4.54*	3.11	5.21	3.78	3.77	2.45	**6.00**	3.44
ASP	4.48	3.08	**5.84**	3.32	4.96*	3.54	**5.62**	4.90	4.90*	4.53	**5.55**	4.05
IDD	3.00	3.19	4.36	3.32	4.75*	3.94	4.92	3.11	4.31*	3.07	4.38	2.79
DS	2.82	2.84	4.00	3.04	4.21*	3.51	4.74	3.03	4.50*	3.18	4.75	3.05
TS	4.62	2.69	6.38*	3.85	4.36*	2.57	5.23	3.95	4.58*	3.12	**5.58**	3.42
ED	3.30	2.67	5.60	3.17	4.25*	2.66	2.75	1.58	2.14	2.48	3.57	2.15
CP	5.66*	3.08	6.78*	2.33	5.62*	3.16	**5.50**	2.33	6.75*	5.19	**6.75**	2.87
SDD	3.67	1.94	4.56	3.39	5.63*	3.54	4.75	4.40	4.86*	3.85	4.57	4.24

*ADHD* attention deficit hyperactivity disorder, *RD* rare disorder, *AUT* autism, *ECD* emotional and conduct disorder, *SD* somatic disorder, *ASP* asperger syndrome, *IDD* intellectual disability disorders, *DS* down syndrome, *TS* tourette’s syndrome, *ED* eating disorders, *CP* cerebral palsy, *SDD* specific developmental disorders. Numbers in bold are the four highest scores on each dependent variable. The numbers also marked with * indicate value at or above the level of “slightly raised” according to British norms

Regression analysis with family economy and parent education as predictor of sibling mental health showed that family economy predicted sibling mental health reported by mothers and father, both externalizing and internalizing problems. Fathers’ education predicted sibling mental health, externalizing problems. Regression analyses with all the diagnostic categories as predictors, independent of whether they were a primary, secondary, or tertiary diagnosis for the six mental health outcomes (externalizing and internalizing scores reported by siblings, and their mothers and fathers) were ran. However, due to too some very small and uneven distribution between diagnostic groups, the individual effect of each predictor (diagnostic groups) on the dependent variable (mental health) were not possible to identify. Hence, to answer the study questions, we selected the five most frequent *primary* diagnoses in the further analysis. The regression analyses with the most frequent primary diagnostic categories (ADHD, rare disorder, Asperger syndrome, autism, and Down syndrome) in the study population for all six mental health scores (sibling, mother and father-rated internalizing and externalizing problems) showed that no single primary diagnosis significantly predicted any of the sibling mental health measures in any of the regression models. However, the disorder severity reported by the fathers significantly predicted internalizing problems reported by the siblings themselves (Table 4) and internalizing and externalizing problems for siblings reported by fathers (Table 5). The disorder severity reported by mothers significantly predicted sibling internalizing problems also reported by the mothers. Gender was a significant predictor for externalizing problems (mothers’ and fathers’ report), where being a boy (sibling) implied more externalizing problems.

**Table 4:** Regression model predicting siblings’ own report about mental health (N = 200)

Dependent variables	Predictors	Adj R2	B	95%CI	p
SDQ siblings					
		0.05			0.13
Internalizing					
	ADHD		0.41	− 0.81, 1.64	0.50
	Aspergers’ syndrome		0.40	− 1.28, 2.07	0.64
	Autism		1.53	− 0.28, 3.33	0.10
	Rare disorders		− 0.67	− 2.10, 0.75	0.35
	Down syndrome		− 1.85	− 3.84, 0.14	0.07
	DBC-P Mother		− 0.04	− 0.08, 0.00	0.05
	DBC-P Father		0.05	0.01, 0.10	0.02*
	Age		0.19	− 0.14, 0.51	0.25
	Gender		0.73	− 0.64, 2.11	0.29
		0.00			0.42
Externalizing					
	ADHD		0.74	− 0.50, 1.97	0.24
	Aspergers’ syndrome		− 0.17	− 1.86, 1.52	0.84
	Autism		1.41	− 0.41, 3.23	0.13
	Rare disorders		− 0.54	− 1.98, 0.90	0.46
	Down syndrome		− 1.29	− 3.30, 0.72	0.21
	DBC-P Mother		− 0.01	− 0.05, 0.03	0.57
	DBC-P Father		0.02	− 0.03, 0.06	0.40
	Age		0.13	− 0.19, 0.46	0.42
	Gender		− 1.06	− 2.45, 0.33	0.13

*SDQ* the strengths and difficulties questionnaire, *SDQ* sibling internalizing/externalizing = siblings’ own scores on the internalizing scale/externalizing problem scale, *ADHD* attention deficit hyperactivity disorder, *DBC-P* the developmental behavior checklist the parent/carer version

^*^Significant at the p < .05-level. Adj. R^2^ = Adjusted R^2^. B = Unstandardized Beta. CI = 95% confidence intervals for unstandardized coefficients

**Table 5:** Regression model predicting siblings´ mental health reported by mothers and fathers (N = 200)

	SDQ Mothers				SDQ Fathers				
Dependent variables	Predictors	Adj R2	B	95%CI	p	Adj R2	B	95%CI	β
		0.07			0.15	0.61			0.06*
Internalizing									
	ADHD		0.12	− 0.84, 1,07	0.81		− 0.08	− 1.22, 1.05	0.89
	Asperger’s syndrome		0.04	1. 21, 1.29	0.95		− 0.30	− 1.72, 1.11	0.67
	Autism		0.64	− 0.53, 1.81	0.28		0.30	− 1.20, 1.80	0.69
	Rare disorders		− 0.00	− 1.21, 1.29	0.10		− 0.24	− 1.55, 1.07	0.71
	Down syndrome		− 0.53	− 1.94, 0.88	0.46		0.16	− 1.67, 1.10	0.86
	DBC-P		0.03	0.00, 0.05	0.02*		0.05	0.03, 0.08	< 0.00*
	Age		0.67	− 0.18, 0.31	0.56		0.03	− 0.27, 0,33	0.84
	Gender		− 0.86	− 1.87, 0.14	0.09		0.02	− 1.22, 1.27	0.97
		0.09			0.04	0.08			0.02*
Externalizing									
	ADHD		0.42	− 0.57, 1.40	0.40		0.30	− 0.76, 1.37	0.58
	Asperger’s syndrome		0.43	− 0.86, 1.72	0.51		1.00	− 0.32, 2.33	0.14
	Autism		0.63	− 0.57, 1.83	0.30		− 0.67	− 2.07, 0.73	0.35
	Rare disorders		− 0.44	− 1.59, 0.72	0.46		− 0.63	− 1.86, 0.59	0.31
	Down syndrome		− 0.25	− 1.70, 1.20	0.74		0.24	− 1.47, 1.96	0.78
	DBC-P		0.01	− 0.01, 0.03	0.42		0.04	0.01, 0.06	0.01*
	Age		− 0.08	− 0.33, 0.17	0.53		− 0.18	− 0.45, 0.10	0.20
	Gender		− 1.68	− 2.70, − 0.64	0.00*		− 1.09	− 2.25, 0.08	0.07*

## Discussion

We investigated whether sibling mental health was associated with single diagnostic categories and/or by disorder severity in the child with chronic disorder. Due to the small sample size per diagnosis and skewed distribution between groups, we focused on the five largest diagnostic groups of primary diagnoses to examine if any of the single diagnostic categories predicted sibling mental health and/or if symptom severity was a significant predictor. None of the primary diagnoses predicted any of the reports about sibling mental health. Thus, we did not find that siblings´ mental health is explained by disorder specific issues. On the contrary, we found support for the transdiagnostic explanation about what impacts sibling mental health the most, as the disorder severity was a significant predictor in the regression models. We found that fathers’ report about disorder severity predicted both siblings’ own report about their mental health and the fathers’ report about siblings’ mental health while mothers’ report about symptom severity only predicted her report about siblings’ internalizing problems. This is in line with results from other studies which have found only moderate correlations between mother – father ratings about sibling mental health (Griffith et al., 2014). We conclude that research is needed to understand the clinical benefits of gathering data about sibling adjustment from more than one parent in the family.

The findings in the current study underpins this need. Furthermore, finding that the fathers’ reports are better aligned with siblings’ own report than the mothers’, indicate that they are at least as important informants about siblings as the mothers. A possible explanation for the findings on fathers’ reports in this study may be that mothers tend to spend more time caring for the child with a chronic disorder (Uribe-Morales et al., 2021) and possibly leaving more responsibility for the siblings on fathers. The finding in the current study also fits with a finding from another sibling study were siblings’ self-reported psychosocial adjustment was more in line with the father-reports than the mother-reports (Fredriksen et al., 2023).

The regression analysis of family economy and parents’ education level showed that both of these factors were significantly predicting sibling mental health reported by the parents themselves, but not the sibling reported mental health. Father education predicted his report about sibling externalizing mental health. These findings are in line with other studies of siblings’ mental health (Wolff et al., 2022) and studies on children’s mental health in general (e.g., Badini et al., 2024; Peverill et al., 2021). These factors are most likely in the causal pathway of sibling mental health but were not the primary focus of this study. The research questions of this study focused on characteristics of the child with a chronic disorder and its impact on sibling mental health. The well-documented effect of socioeconomic status was therefore not further emphasized in the interpretation of the results of this study.

The results from this study indicate that disorder severity may be the best indicator of which siblings should be targeted for intervention. The common disorder severity factors e.g., communication disturbance, antisocial behavior, social difficulties and how these symptoms affect the siblings and the family system, should be the focus in intervention programs. The finding that disorder severity is an important predictor of sibling mental health is in line with findings in the most resent reviews (Pinquart, 2023; Wolf et al., 2022). However, the reviews also found that the predictors of sibling mental health are composed of several factors. Some studies in the reviews showed that certain disorders may have more negative impact on sibling mental health than others. Pinquart’s meta-analysis (2023) found that both the kind of disorder and its intrusiveness were some of the moderators in the included studies that examined sibling mental health. The result from the regression analyses in the current study find support only for the latter. However, in the descriptive analysis of the level of sibling mental health problems associated with each diagnostic category (Table 3), there are some diagnostic categories that are associated with elevated scores across informants and on both internalizing and externalizing mental health problems. Those who are siblings of children with ADHD, autism, Tourette’s syndrome, and cerebral palsy, show elevated scores across three or more of the six outcome variables. One explanation for this finding may be the heritability of ADHD, autism, and Tourette’s syndrome. ADHD has a hereditability of about 80% (Faraone & Larson, 2019). Thus, the likelihood of having more than one person in the family with the same condition is high and even though the siblings in this study did not meet the diagnostic criteria for ADHD, they may have displayed subclinical levels of symptoms (Andersson Konke et al., 2023; Kleppesto et al., 2024). Hence, their higher (worse) mental health symptom score may reflect hereditary factors rather than the fact that they are a sibling of a child with chronic disorder. However, previous studies have found that siblings of children with ADHD report lower well-being, even when controlling for siblings’ own ADHD symptoms (Peasgood et al., 2016). This could be partially due to parents in families with a child with ADHD being more likely to have ADHD traits themselves and further influence the family dynamics and parents’ ability to cope with a demanding family situation (Carr-Fanning & McGuckin, 2022). The parenting functioning within a family has a significant impact on children´s development and health (Zimmer-Gembeck et al., 2022). Parents´ own difficulties may be an important factor to consider in the development of effective interventions for siblings and may be a reason to custom design interventions according to specific diagnoses. Even though ADHD has a prevalence of around 5% (Fast et al., 2024; Willcutt, 2012), it is a condition that can be difficult for others to identify in children and adolescents. The lesser degree of visibility in ADHD than conditions such as rare disorders may lead to less support, less understanding from others, and thus a greater burden on the sibling. Tourette’s syndrome is also a strongly familial and heritable disorder (Mataix-Cols et al., 2015). Thus, the findings in this study may be explained by the same factors as in ADHD, such as heritability or the presence of symptoms in siblings other than the diagnosed child. However, Tourette’s syndrome is a considerably more visible condition than ADHD and may also lead to emotional burden in form of shame and feeling of embarrassment.

The siblings of children with autism are also of those who show the most mental health problems in this sample. As is the case for ADHD, autism is to some extent hereditary (Tick et al., 2016). Hence, the probability that other family members have autism is higher than in families without autism families (Happé & Frith, 2020; Martini et al., 2024). Thus, the predictive value of autism found in the current study may have been an effect of the fact that family members share autsim-traits. The core traits in autism are difficulties with social interaction and deficit of verbal and nonverbal communication, as well as repetitive behaviors and restricted interests (ICD-10). These difficulties may have a considerable impact on family functioning (i.e., the interaction between family members and how the family operates) where difficulties have shown to be highly correlated with more caregiver demand and less resources (Desquenne Godfrey et al., 2023; Garrido et al., 2020; Karst & Van Hecke, 2012). The finding of more problems in siblings of children with autism than in other diagnostic groups and/or siblings of children with no disorders are found in several studies (Shivers et al., 2019). The meta-analysis of Shivers et al. (2019) found significantly more negative outcomes on several areas (psychological functioning, beliefs, sibling relationship beliefs about disability, anxiety and depression symptoms) that seems connected to the burden of being a sibling of a child with autism as well as hereditability. Thus, the impact on sibling mental health may arise through both genetic and environmental pathways.

As cerebral palsy is a condition that is mainly caused by perinatal risk factors like preterm birth, perinatal infection, acidosis, or asphyxia this condition rarely occurs in more than one family member. However, cerebral palsy may be a very complex condition with not only motor or movement problems but also cognitive dysfunction, seizures, behavioral or emotional problems, and speech as well as hearing impairment (Vitrikas et al., 2020). The explanation of the fact that the siblings of children with CP in the sample of the current study are reported as having the most mental health problems, may be a result of the severity or complexity of these children’s´ condition.

Across all the prediction models we controlled for the siblings´ age and gender and found that sibling gender was a significant predictor in the models with parent reported SDQ, where being a sibling boy was associated with more externalizing problems. Age was not a significant predictor in any of the regression models. The results align with findings in other studies when it comes to gender. Studies suggest that gender influences the caring role of siblings, where older females in families with a higher child to parent ratio take on more caring responsibilities (Boyle et al., 2023; Hilario, 2022) and boys tend to react to stress with externalizing symptoms and girls with internalizing problems (Lavoie et al., 2019). Some previous studies have found that younger siblings are less vulnerable than older siblings (Vermaes et al., 2012). However, sibling age was not a significant predictor in this study. Thus, age as a single factor was not found to be explanatory for how siblings’ mental health is affected by the chronic disorder. There are both protective and risk factors associated with being a young sibling as well as being an older sibling; siblings of children with chronic disorders have described their sibling relationship differently depending on age, where arguing and conflicts lessened when age increased or when siblings move out from home (Hamwey et al., 2019; Webster, 2018). Being an older sibling may imply more responsibilities and tasks (Boyle et al., 2023; Hilario, 2022). They may also have more insight into negative information about the chronic disorder and thus worry more than younger children (Malcolm et al., 2014). However, older siblings are also less dependent on their parents and may be less affected by differential parental attention. Moreover, age in itself may be subordinated by sibling order, number of children in the family, spacing and/or personality, and other factors like family functioning.

## Strengths and Limitations

This study has several strengths. First, the sample was fairly large and allowed for comparison of the most frequent primary diagnostic categories. Second, different diagnoses were included in the same study. This makes the comparison between diagnostic categories more easily interpretable than if they had been recruited from different studies. Second, most research on sibling mental health is based on mother report (Giallo et al., 2012; Pinquart, 2023). We included father report and sibling self-report in the current study and are thus contributing to knowledge about how siblings perceive their own experiences. Furthermore, the siblings´ self-report can be compared to parent reports in the same sample. Third, a measure of disorder severity was included across all diagnostic categories and allowed for an analysis of whether disorder severity predicted sibling mental health better than the diagnostic categories within the same sample.

This study also has limitations. First, there was a skewed distribution of number of children in the different diagnostic categories and this effected the possibility to include all diagnostic categories in the regression analysis. However, the skewed distribution did reflect the prevalence of the different disorders in the population, with ADHD being the most common child psychiatric disorder (Fast et al., 2024). Further, three of the primary diagnoses (ADHD, autism, Asperger syndrome) included in the regression analyses may be seen as more related with each other than with the fourth and fifth category (rare disorders and Down syndrome). Hence, the comparison between the three diagnostic categories that are etiologically and conceptually related may be questioned. Most participants were European-White, on average highly educated and hence may not be representative across families of children with chronic disorders. The DBC-P is a well-established questionnaire specially designed to capture emotional and conduct problems in children with developmental and intellectual disabilities. However, DBC-P does not capture emergency situations due to sudden deterioration of the chronic disorder itself or additional complications like infections or respiratory arrest which may occur in the children with rare disorders. Sudden and frightening experiences like these may have a negative impact on siblings which is not captured in this study. Thus, the impact on the mental health of siblings of children with rare disorders may have been underestimated.

## Conclusions and Recommendations for Future Research

The main finding of this study was that disorder severity predicted both sibling- and parent-reported sibling mental health and may, therefore, be an important indicator of which siblings may be at risk for developing mental health problems and are in need of interventions. Further, the results of this study support a transdiagnostic understanding of impact on sibling mental health. Interventions should therefore focus on common factors like communication disturbance, antisocial behavior, social difficulties and how these symptoms affect the siblings and the family system. One should strive to include fathers in research and in interventions as it seems as they may have a significant role toward siblings of children with a chronic disorder. The current study indicates that some diagnoses may have more impact on siblings than others. Research should strive to examine certain vulnerable groups and design interventions that are tailored specifically to improve diagnoses specific challenges like in families with a child with ADHD where parents may have their own difficulties with emotional regulation, planning and organizing.
